# Clinical and genomic characterization of carbapenem-resistant *Enterobacterales* bloodstream infections in patients with hematologic malignancies

**DOI:** 10.3389/fcimb.2024.1471477

**Published:** 2024-09-26

**Authors:** Yi Chen, Jiangqing Huang, Luyan Dong, Binbin Xu, Lei Li, Zhichang Zhao, Bin Li

**Affiliations:** ^1^ Fujian Medical University Union Hospital, Fujian Institute of Hematology, Fujian Provincial Key Laboratory on Hematology, Fuzhou, China; ^2^ Department of Clinical Laboratory, Fujian Medical University Union Hospital, Fuzhou, China; ^3^ Department of Clinical Laboratory, Fujian Medical University Union Hospital Pingtan Branch, Fuzhou, China; ^4^ Department of Pharmacy, Fujian Medical University Union Hospital, Fuzhou, China

**Keywords:** carbapenem-resistant *Enterobacterales*, hematological malignancies, clinical characteristics, whole-genome sequencing, prognosis

## Abstract

**Background:**

Carbapenem-resistant *Enterobacterales* (CRE) bloodstream infections (BSIs) pose a significant risk to patients with hematologic malignancies, yet the distinct features and outcomes of these infections are not thoroughly understood.

**Methods:**

This retrospective study examined the characteristics and clinical outcomes of patients with *Enterobacterales* BSIs at the Hematology Department of Fujian Medical University Union Hospital from 2018 to 2022. Whole-genome sequencing was conducted on 45 consecutive CRE BSI isolates during this period.

**Results:**

A total of 301 patients with *Enterobacterales* BSIs were included, with 65 (21.6%) cases of CRE and 236 (78.4%) cases of carbapenem-susceptible *Enterobacterales* (CSE). CRE infections accounted for 16.9% to 26.9% of all *Enterobacterales* BSIs, and carbapenem-resistant *Klebsiella pneumoniae* (CRKP) was the predominant strain. The most frequent sequence type (ST) and carbapenemase among CRKP were ST11 (68.6%) and *bla*KPC-2 (80.0%), respectively. Perianal infections, multiple infection foci, and a history of multiple hospitalizations, ICU stays, and prior CRE infections were identified as risk factors for CRE BSIs. Patients in the CRE group experienced significantly higher proportions of infection-related septic shock (43.1% vs. 19.9%, P < 0.0003) and 30-day all-cause mortality (56.9% vs. 24.6%, P < 0.0001) compared to those in the CSE group. Patient’s age and disease subtypes, strain subtypes, and antimicrobial treatment regimens significantly influenced survival in patients with CRE BSIs.

**Conclusions:**

CRE BSIs are a frequent complication in patients with hematological malignancies undergoing treatment and are associated with poor survival rates. A comprehensive understanding of risk factors and ongoing surveillance of prevalent strains are essential for the effective management of these infections.

## Introduction

Over the past several decades, carbapenem antibiotics have emerged as a critical line of defense against multidrug-resistant (MDR) gram-negative bacterial infections (Hawkey and Livermore, 2012). However, the widespread use of carbapenems has been accompanied by a troubling surge in carbapenem-resistant organisms (CROs) (Bonomo et al., 2018; Nordmann and Poirel, 2019), including carbapenem-resistant *Enterobacterales* (CRE), *Pseudomonas aeruginosa* (CRPA), and *Acinetobacter baumannii* (CRAB) (Potter et al., 2016; Lutgring, 2019; Tomczyk et al., 2019; Tenover et al., 2022). Patients with hematological malignancies, who often experience immunodeficiency due to chemotherapy and transplantation, represent a particularly high-risk population for CRO infections (Johnson and Boucher, 2014; Wang et al., 2023; Delanote et al., 2024).

Bloodstream infections (BSIs) are among the most severe types of bacterial infections, with the potential to be life-threatening (Kochanek et al., 2019). Although advancements in antibacterial therapy, including the use of broad-spectrum agents, have mitigated BSI mortality rates, the rising incidence of CROs, particularly CRE, poses a significant threat to these patients (Satlin et al., 2014; Brink, 2019; Nordmann and Poirel, 2019; Kern and Rieg, 2020; Amanati et al., 2021). The high mortality rates associated with CRE BSIs in hematological patients highlight the need for heightened awareness and management strategies among hematologists (Satlin et al., 2016).

Despite the importance of CRE BSIs in this patient population, there is a lack of comprehensive research on the clinical and microbiological characteristics of these infections. This gap in knowledge leaves hematologists with insufficient data to effectively manage CRE BSIs. To address this void, we conducted a retrospective analysis of patients with *Enterobacterales* BSIs at the Hematology Department of a tertiary care hospital in Fuzhou, China. We retrieved data on all patients with *Enterobacterales* BSIs from 2018 to 2022 and performed whole-genome sequencing (WGS) on CRE-BSI isolates from this period. Our findings aim to provide a practical understanding of the clinical and microbiological features of CRE BSIs in hematologic malignancy patients, which may inform more effective management strategies for these complex infections.

## Methods

### Patient population

This investigation was carried out within the Hematology Department of Fujian Medical University Union Hospital, Fuzhou, China, a major referral center with 240 beds dedicated to hematological conditions. The study population consisted of patients with hematological malignancies who were diagnosed with *Enterobacterales* BSIs between January 1, 2018, and December 31, 2022. The research protocol was approved by the Institutional Review Board of Fujian Medical University, and all procedures adhered to the ethical principles outlined in the 1964 Declaration of Helsinki.

### Definitions

In this study, a BSI caused by *Enterobacterales* was defined as the isolation of one or more of these bacteria in a single blood culture bottle during the study period. The term “carbapenem resistance” was used to describe bacteria that exhibited a minimum inhibitory concentration (MIC) of meropenem and/or imipenem equal to or greater than 4 mg/mL (Mermel et al., 2009). Septic shock was diagnosed based on clinical criteria, including hypotension requiring vasopressor therapy to maintain a mean blood pressure of 65 mm Hg or higher after appropriate fluid resuscitation (Durante-Mangoni et al., 2019). Neutrophil was defined as a neutrophil count less than 0.5 × 10^9^/L in the peripheral blood.

### Record review

**Figure 1:**
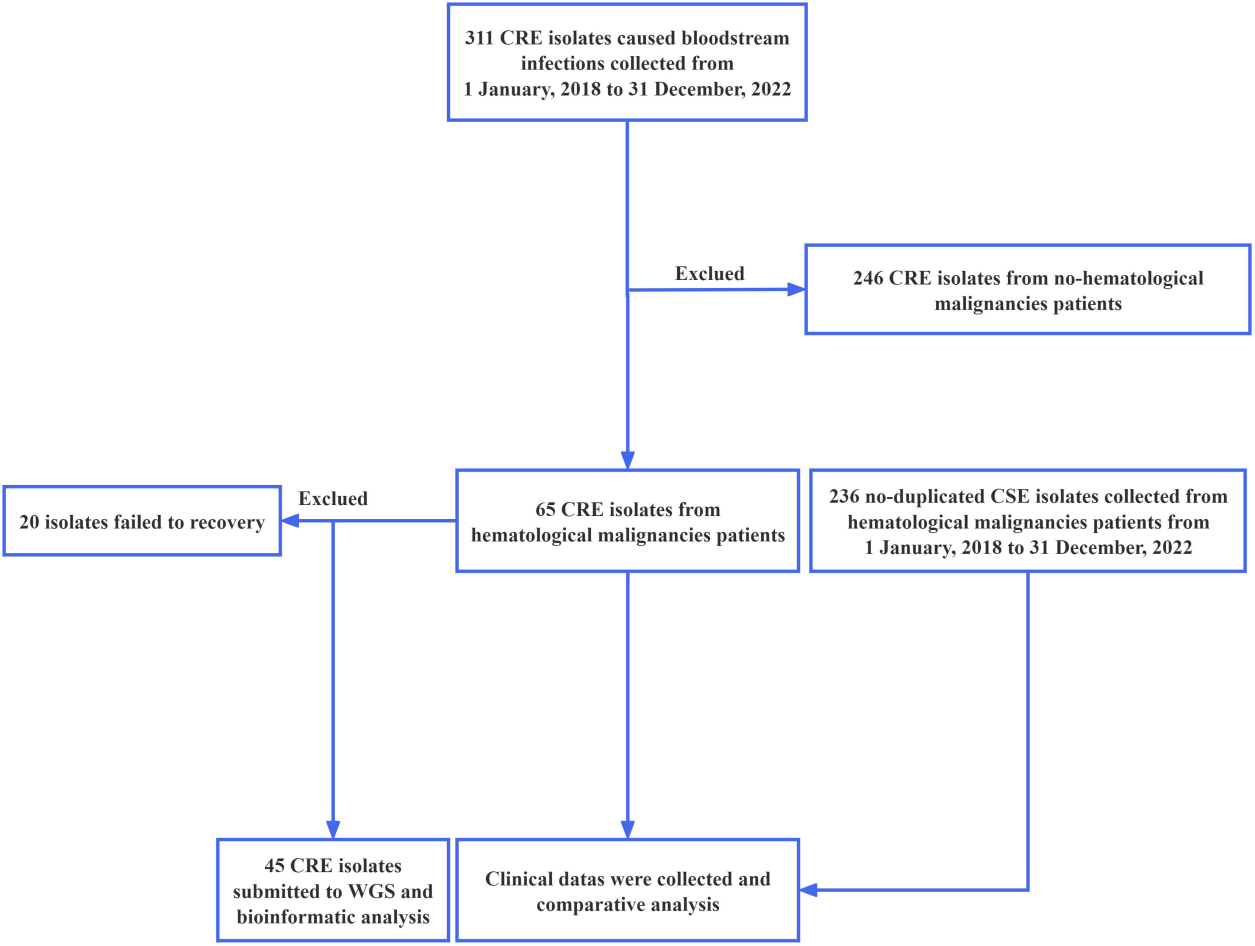
Flowchart illustrating the design and procedure of this study. CRE, Carbapenem-resistant *Enterobacterales*.

The clinical and microbiological data were meticulously extracted from the medical records of the patients. A detailed summary of the data collection process is presented in **Figure 1**. A team of clinicians carefully reviewed each case to ensure that the primary infection sites met the criteria established by the National Healthcare Safety Network. Catheter-related bloodstream infections (CR-BSIs) were defined in accordance with the guidelines set by the Infectious Diseases Society of America (IDSA) (Shankar-Hari et al., 2016). These CR-BSIs were excluded from the study. In instances where no primary infection site met the specified criteria, the infection source was categorized as “unidentified”.

### Bacterial isolates

The bacterial isolates obtained from blood samples were cultivated on blood agar plates (Autobio Diagnostics CO.,Ltd, China). The bacterial species were identified using Bruker Biotyper (Microflex LT/SH Smart) MALDI-TOF MS (Bruker Daltonics GmbH, Bremen, Germany). Antimicrobial susceptibility testing was conducted using the Vitek-2 system (Vitek-AST-GN16) (BioMerieux, France), following the guidelines provided by the Clinical and Laboratory Standards Institute (CLSI, 2022). These methodologies were implemented in the Clinical Microbiology Laboratory of Fujian Medical University Union Hospital. All cases of *Enterobacterales* isolated from 2018 to 2022 were included into this study. For patients from whom many CRE were isolated, only a representative isolate was included in our study to avoid redundancy.

### Genome sequencing and analysis

Genomic DNA from the CRE isolates was extracted using the TIANamp Bacteria DNA Kit (Tiangen, Beijing). Sequencing was conducted on the Illumina NovaSeq platform at Shanghai Personal Biotechnology Co., Ltd (Shanghai, China) in a paired-end mode with an insert size of 2 × 150 base pairs. The depth of coverage varied from 224-fold for 10,459,852 reads to 292-fold for 10,256,762 reads, with an average depth of coverage of approximately 260-fold. Gene content analysis was performed using tools provided by the Center for Genomic Epidemiology (https://cge.cbs.dtu.dk/services/). This included the identification of acquired resistance genes and known chromosomal mutations associated with antibiotic resistance using the ResFinder tool (Yu et al., 2019). Plasmid types were identified using PlasmidFinder (Zankari et al., 2012), and virulence genes were identified using VirulenceFinder (Carattoli et al., 2014). Multilocus-sequence typing (MLST) was conducted on the MLST website (https://cge.food.dtu.dk/services/MLST/). The genetic environment of the *bla*KPC-2 and *bla*NDM-5 genes was predicted using the RAST tool (https://rast.nmpdr.org/rast.cgi). Core-genome alignments were utilized for phylogenetic tree reconstruction using maximum likelihood estimation.

### Statistical analysis

Differences between patient subgroups were statistically evaluated using the chi-square test, t-test, or nonparametric test, as appropriate for the nature of the data. GraphPad Prism version 9.5 was employed for all statistical analyses. A P value of less than 0.05 (two-tailed) was considered to indicate a statistically significant difference.

## Results

### Patient characteristics

From January 1, 2018, to December 31, 2022, a total of 301 patients with *Enterobacterales* BSIs were enrolled in the study, including 65 cases (21.6%) with CRE and 236 cases (78.4%) with carbapenem-susceptible *Enterobacterales* (CSE). The patient cohort consisted of 172 males (57.1%) and 129 females (42.9%), with a wide age range from 1 to 80 years, and a median age of 46 years. The analysis of demographic data showed no significant differences in age, gender, disease subtypes, or the proportion of patients with peripherally inserted central catheters (PICCs) between patients with CRE and CSE BSIs (**Table 1**).

**Table 1:** Characteristics of patients with *Enterobacterales* BSIs.

Parameters	All (N=301)	CRE (N=65)	CSE (N=236)	P-value
Male sex, n (%)	172 (57.1)	40 (61.5)	132 (55.9)	0.4799
Median age, year (range)	46 (1-80)	42 (2-76)	47 (1-80)	0.2804
Types of diseases, n (%)				
Myeloid leukemia	134 (44.5)	31 (47.7)	103 (43.6)	0.5757
Lympholastic leukemia	90 (29.9)	15 (23.1)	75 (31.8)	0.2209
lymphoma	46 (15.3)	13 (20.0)	33 (14.0)	0.2451
Multiple myeloma	20 (6.6)	5 (7.7)	15 (6.4)	0.7783
Myelodysplastic syndromes	11 (3.7)	1 (1.5)	10 (4.2)	0.4668
Peripherally inserted central catheter, n (%)	289 (96.0)	62 (95.4)	227 (96.2)	0.7262

P-value, comparison between CRE and CSE bloodstream infection. BSI, bloodstream infection; CRE, carbapenem-resistant Enterobacterales; CSE, carbapenem-susceptible Enterobacterales.

### Infection sources and pathogens

The details of infection sources for the patients with *Enterobacterales* BSIs are summarized in **Table 2**. The respiratory and digestive tracts were the most common sites of infection, accounting for 62.1% and 24.6% of cases, respectively. Notably, patients with CRE BSIs had a significantly higher proportion of perianal infections (29.2%) and multiple infection foci (52.3%) compared to those with CSE BSIs (13.1% and 38.1%, respectively). Additionally, the proportion of patients with an unidentified infection source was significantly lower in the CRE group (3.1%) compared to the CSE group (14.4%). The primary pathogens associated with *Enterobacterales* BSIs were carbapenem-resistant *Escherichia coli* (CREC), which accounted for 55.1% of cases, while carbapenem-resistant *Klebsiella pneumoniae* (CRKP) was the predominant CRE isolate, representing 69.2% of all CRE BSIs.

**Table 2:** Infection sources and pathogens in patients with *Enterobacterales* BSI.

Parameters	All (N=301)	CRE (N=65)	CSE (N=236)	P-value
Infection sources, n (%)				
Respiratory tract	187 (62.1)	43 (66.2)	144 (61.0)	0.4740
Gastrointestinal tract	74 (24.6)	20 (30.8)	54 (22.9)	0.1965
Urinary tract	15 (5.0)	3 (4.6)	12 (5.1)	>0.9999
Perianal	50 (16.6)	19 (29.2)	31 (13.1)	0.0041
Multiple	124 (41.2)	34 (52.3)	90 (38.1)	0.0465
Unidentified	56 (18.6)	6 (9.2)	50 (21.2)	0.0168
Types of bacteria, n (%)				
*Klebsiella pneumoniae*	139 (46.2)	45 (69.2)	94 (39.8)	<0.0001
*Escherichia coli*	144 (47.8)	14 (21.5)	130 (55.1)	<0.0001
Others	18 (6.0)	6 (9.2)	12 (5.1)	0.2372

BSI, bloodstream infection; CRE, carbapenem-resistant Enterobacterales; CSE, carbapenem-susceptible Enterobacterales.

### Microbiological characteristics

**Figure 2:**
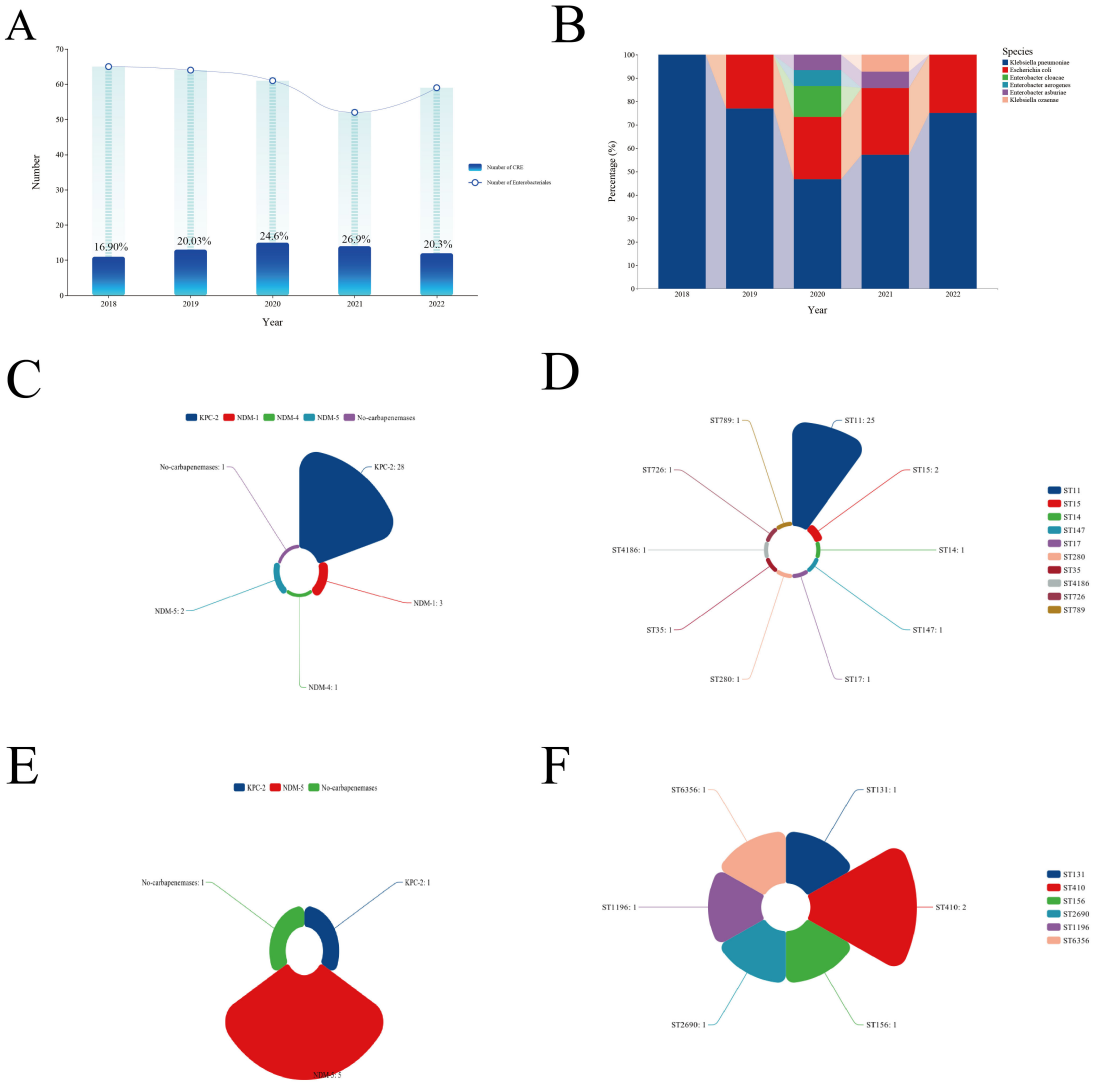
Clinical and microbiological characteristics of CRE BSIs. **(A)** Incidence of CRE BSIs over the study period. **(B)** Types of CRE strains isolated. **(C)** Types of carbapenemases detected in CRKP strains. **(D)** Predominant STs of CRKP. **(E)** Types of carbapenemases detected in CREC strains. **(F)** Predominant STs of CREC. CRE, Carbapenem-resistant *Enterobacterales*; BSI, Bloodstream infection; CRKP, Carbapenem-resistant *Klebsiella pneumoniae*; ST, Sequence type; CREC, Carbapenem-resistant *Escherichia coli*.

The incidence of CRE infections among patients with *Enterobacterales* BSIs varied between 16.9% and 26.9% from 2018 to 2022, with the highest rate recorded in 2021 (**Figure 2A**). CRKP was the predominant CRE isolate identified during this period (**Figure 2B**). Genome sequencing was conducted on 35 CRKP cases, seven CREC cases, two carbapenem-resistant *Enterobacter cloacae* cases, and one carbapenem-resistant *Klebsiella michiganensis* case. Genetic analysis revealed that the predominant sequence type (ST) of CRKP was ST11, accounting for 68.6% of all cases, while CREC displayed a diverse range of ST types (**Figures 2C, D**). Enzymatic profiling indicated that 80% of CRKP carried the *bla*KPC-2 gene, and 71.4% of CREC carried the *bla*NDM-5 gene (**Figures 2E, F**).

### Genomic diversity

**Figure 3:**
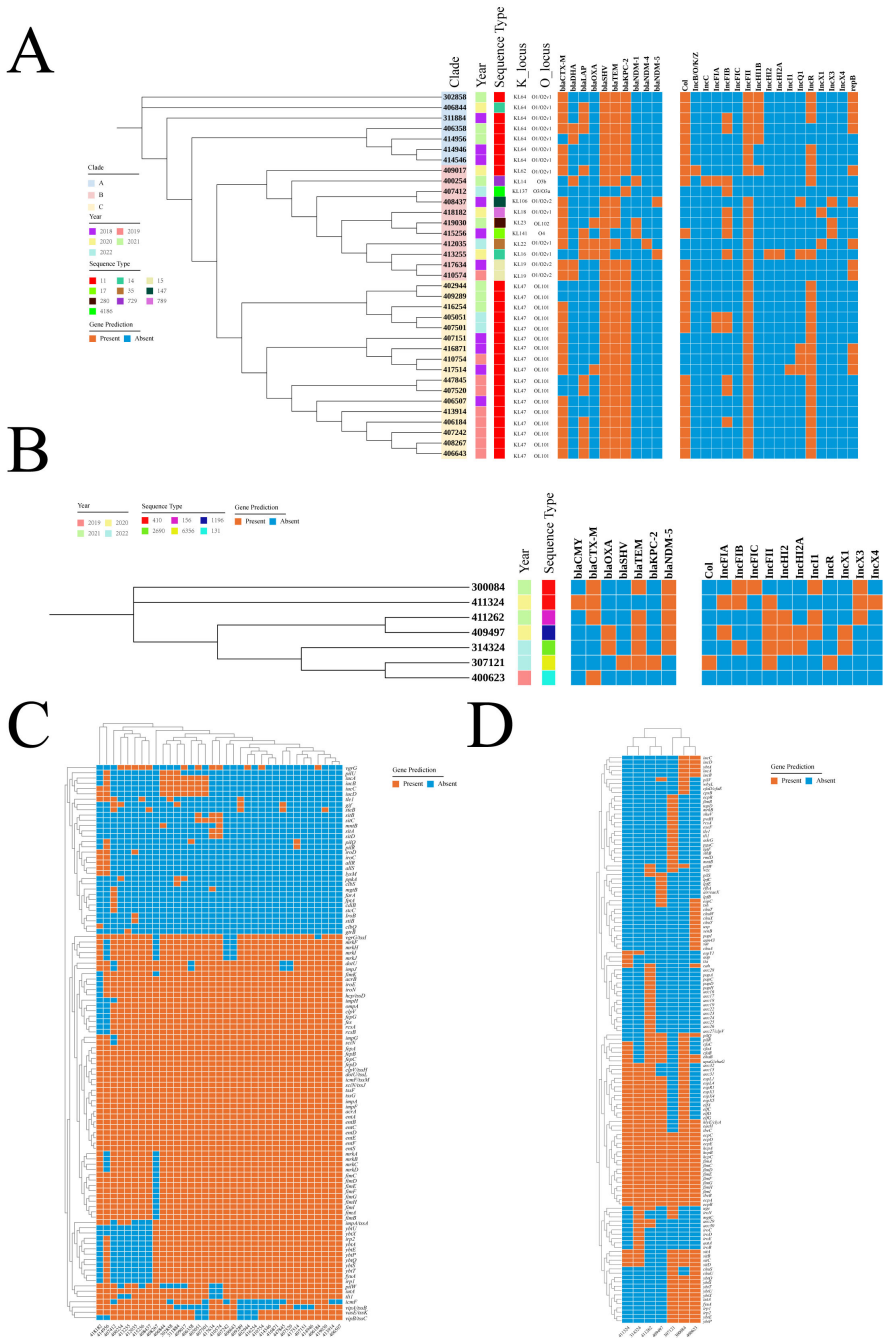
Genomic characteristics of CRKP and CREC. **(A)** Distribution of years, STs, K loci, O loci, and drug resistance genes in CRKP. **(B)** Distribution of years, STs, K loci, O loci, and drug resistance genes in CREC. **(C)** Distribution of virulence genes in CRKP. **(D)** Distribution of virulence genes in CREC. ST, Sequence type; CRKP, Carbapenem-resistant *Klebsiella pneumoniae*; CREC, Carbapenem-resistant *Escherichia coli*.

As it was showed at **Figure 3A**, genetic background analysis of CRKP identified three distinct clades. The ST11-KL47-OL101 and ST11-KL64-O1/O2v clones were the predominant ones, with the ST11-KL47-OL101 clone possibly being involved in nosocomial infections in 2019. Additionally, 97.1% of CRKP strains carried multiple β-lactamase genes and plasmids. For CREC, analysis did not exhibit distinct clades, suggesting a high possibility of dissemination and transmission (**Figure 3B**). The distribution of virulence genes in CRKP and CREC was summarized in **Figures 3C, D**, respectively. Although these strains harbored a considerable number of virulence genes, none of the genes specifically associated with hypervirulent *Klebsiella pneumoniae*, including *rmpA/rmpA2*, *peg-344*, *iroB*, and *iucA*, which are particularly associated with CRKP, were detected.

### Genetic environments of *bla*KPC-2 and *bla*NDM-5

**Figure 4:**
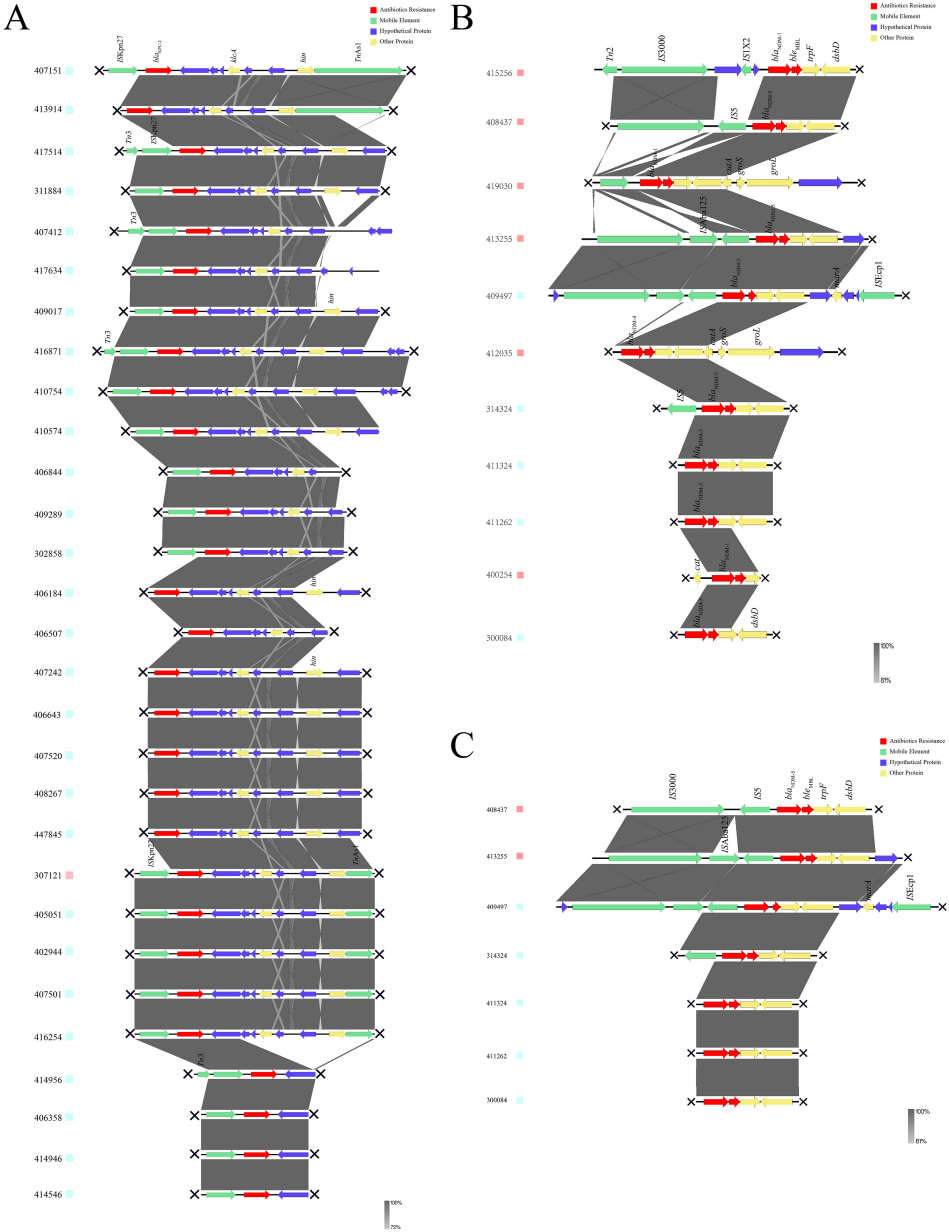
Genetic environments of carbapenemases. **(A)** Genetic environment of *bla*KPC-2. **(B)** Genetic environment of *bla*NDM. **(C)** Genetic environment of *bla*NDM-5. KPC, Klebsiella pneumoniae carbapenemase; NDM, New Delhi metallo-β-lactamase.

The genetic environment upstream and downstream of the *bla*KPC-2 gene was shown in **Figure 4A**, with the core part of the gene environment primarily located between ISKpn27 and/or TnAs1. In **Figure 4B**, the upstream and downstream genetic environment of the *bla*NDM gene was illustrated, revealing significant differences in the genetic environments of different NDM subtypes, suggesting different plasmids or origins for these subtypes. **Figure 4C** depicted the genetic environment of the *bla*NDM-5 gene, which was the predominant subtype of *bla*NDM. The core region of *bla*NDM-5 exhibited a similar genetic environment, predominantly containing the structure of IS5-*bla*NDM-5-*ble*MBL-trpF-dsbD. However, multiple IS sequences were observed near *bla*NDM-5 in some strains, indicating a high possibility of horizontal transfer and dissemination, as indicated by strain numbers 413255 and 409497.

### Risk factors for CRE BSIs

**Figure 5:**
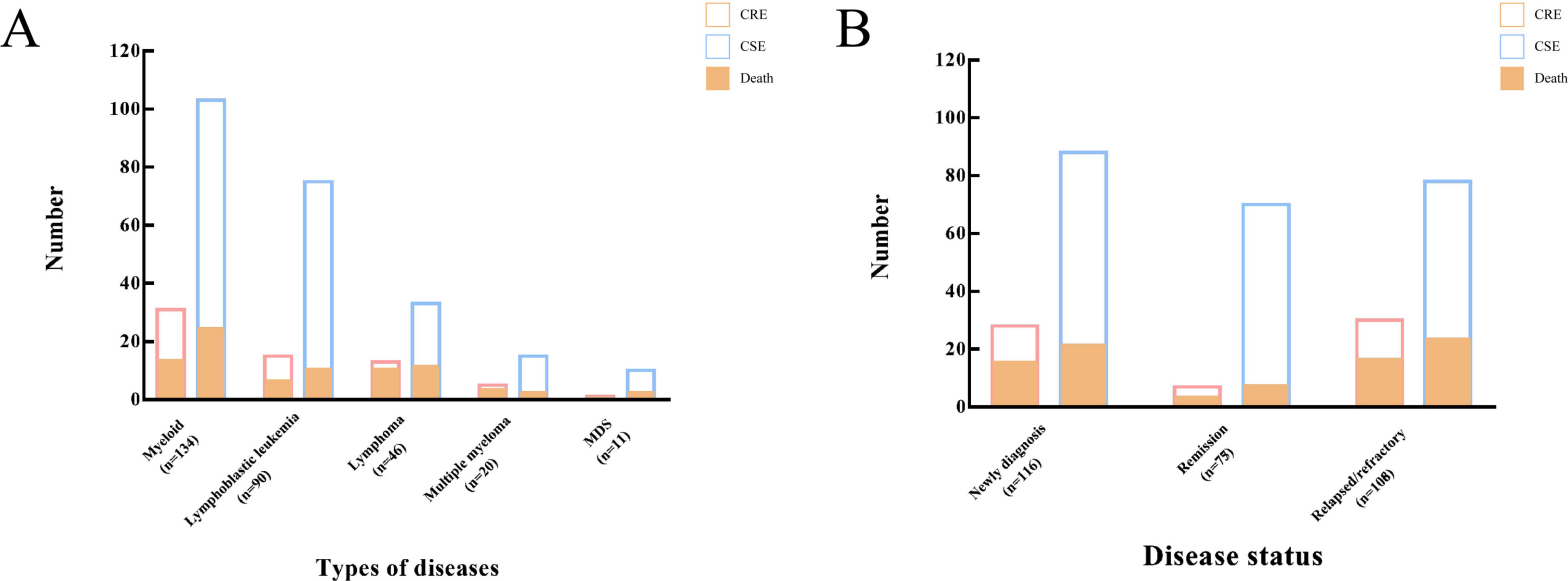
Incidence and mortality of CRE BSIs in patients with hematological malignancies. **(A)** Distribution of CRE BSIs by disease subtype. **(B)** Distribution of CRE BSIs by disease status. CRE, Carbapenem-resistant *Enterobacterales;* BSIs, bloodstream infections.

A total of 34.6% of patients had been treated with carbapenems within the three months preceding sample isolation, with no significant difference between the CRE and CSE groups. Notably, compared to the CSE group, patients in the CRE group had a significantly higher rate of histories of multiple hospitalizations (70.8% vs. 34.3%, P <0.0001), ICU exposure (18.5% vs. 2.1%, P <0.0001), and prior CRE infection (18.5% vs. 3.8%, P = 0.0002). The CRE group primarily consisted of patients with newly diagnosed (43.1%) and relapsed/refractory diseases (46.2%), with a small proportion (10.8%) of patients in remission status (**Figure 5**). In contrast, the CSE group had a relatively even distribution among these categories, with proportions of 37.3%, 33.1%, and 29.7% respectively. Administration of glucocorticoids, transplantation, and immunosuppressants was similar between the CRE and CSE groups. No significant differences were observed in the proportion of patients with neutropenia, or in the levels of C-reactive protein (CRP), procalcitonin (PCT), and interleukin-6 (IL-6) between the two groups. The detailed clinical characteristics of CRE BSIs are summarized in **Table 3**.

**Table 3:** Comparison of clinical characteristics and treatment outcomes between CRE and CSE groups.

Parameters	CRE (N=65)	CSE (N=236)	P-value
History, n (%)			
Multiple hospitalizations	46 (70.8)	81 (34.3)	<0.0001
Exposure to carbapenem antibiotics within 3 months	25 (38.5)	79 (33.5)	0.4644
Exposure to ICU	12 (18.5)	5 (2.1)	<0.0001
CRE infection	12 (18.5)	9 (3.8)	0.0002
Disease status, n (%)			
Newly diagnosed	28 (43.1)	88 (37.3)	0.4719
Remission	7 (10.8)	70 (29.7)	0.0020
Relapsed/refractory	30 46.2)	78 (33.1)	0.0582
Glucocorticoid in chemotherapy regimens, n (%)	33 (50.8)	123 (52.1)	0.8890
HSCT, n (%)	18 (27.7)	60 (25.4)	0.7498
Immunosuppressant after allo-HSCT	16 (24.6)	52 (25.0)	0.7377
Laboratory indicators			
Neutropenia, n (%)	54 (83.1)	213 (90.3)	0.1216
Median CRP (range), mg/ml	101.0 (0.4-408.0)	81.2 (0.5-542.0)	0.3490
Median PCT (range), pg/ml ^1^	1.77 (0.43-100.00)	1.17 (0.30-100.00)	0.5153
Median IL-6 (range), IU/ml ^2^	776.7 (2.8-5000.0)	526.6 (3.5-5000.0)	0.4310
Treatment outcomes, n (%)			
Septic shock	28 (43.1)	47 (19.9)	0.0003
Mortality within 30 days	37 (56.9)	54 (22.9)	<0.0001

CRE, carbapenem-resistant Enterobacterales; CSE, carbapenem-susceptible Enterobacterales. ICU, intensive care unit; HSCT, hematopoietic stem cell transplantation; CRP, C-reactive protein; PCT, procalcitonin; IL-6: interleukin-6. 1. The detection upper limit of PCT is 100 pg/ml in our center; 2. The detection upper limit of IL-6 is 5000 IU/ml in our center.

### Risk factors for in-hospital death

In the CRE group, infection-related septic shock was more common compared to the CSE group (43.1% vs. 19.9%, P = 0.0003), and the all-cause mortality rate within 30 days was significantly higher in the CRE group (56.9% vs. 24.6%, P <0.0001). Age was a factor that influenced mortality, with a rate of 45.5% for those under 46 years and 68.8% for those 46 years or older (P = 0.0804). The disease subtype and status had a significant impact on the occurrence and survival of CRE BSIs in patients with malignant hematologic disorders. For example, the mortality rate for patients with acute leukemia was 45.7%, which was lower than the rate for patients with lymphoma, multiple myeloma (MM), or myelodysplastic syndrome (MDS) (84.2%, P = 0.0056), and no difference was observed between acute myeloid leukemia (AML) and acute lymphoblastic leukemia (ALL).

Additionally, strains carrying *bla*KPC-2 or *bla*NDM-5 were associated with higher mortality rates compared to those without these carbapenemases. The administration of tigecycline and/or polymyxin B could reduce mortality, with the combination therapy of tigecycline and polymyxin B exhibiting the lowest mortality rate at 34.5%. Notably, the mortality rate among patients who developed septic shock was 78.6%, which was significantly higher than the 40.5% mortality rate among those who did not develop septic shock (P = 0.0026). The prognostic factors for patients with CRE BSIs are summarized in **Table 4**.

**Table 4:** Prognostic factors of patients with CRE BSIs.

Parameters	30-day mortality rate, n (%)	P-value
Sex		
Male	23/40 (57.5)	>0.9999
Female	14/25 (56.0)	
Age, years		
<60	25/50 (50.0)	0.0723
≥60	12/15 (80.0)	
Types of diseases		
Myeloid leukemia	14/31 (45.2)	0.0812
Lymphoblastic leukemia	7/15 (46.7)	
Lymphoma	11/13 (84.6)	
Multiple myeloma	4/5 (80.0)	
Myelodysplastic syndromes	1/1 (100.0)	
Disease status		
Newly diagnosed	16/28 (57.1)	0.9993
In remission	4/7 (57.1)	
Relapsed/refractory	17/30 (56.7)	
Types of bacteria, n (%)		
*Klebsiella pneumoniae*	27/45 (60.0)	0.7540
*Escherichia coli*	7/14 (50.0)	
Others	3/6 (50.0)	
Sequence type		
ST11	16/24 (66.7)	0.2111
Others	8/18 (44.4)	
Carbapenemase enzyme		
*bla*KPC-2	18/29 (62.1)	0.4325
*bla*NDM-5	4/7 (57.1)	
Others	2/6 (33.3)	
Antimicrobial treatment regimens		
Without Tigecycline or Polymyxin B	8/10 (80.0)	0.0981
With Tigecycline	23/48 (47.9 )	
With Polymyxin B	16/36 (44.4)	
With Tigecycline and Polymyxin B	10/29 (34.5)	
Septic shock		
Present	22/28 (78.6)	0.0026
Absent	15/37 (40.5)	

CRE, carbapenem-resistant Enterobacterales; BSIs, bloodstream infections.

## Discussion

Patients with malignant hematological diseases are at high risk for BSIs during hospitalization due to their immunodeficiency (van Loon et al., 2017; Zhang et al., 2018). *Enterobacterale*s are the most common Gram-negative bacteria causing BSIs, and CRE has emerged as a frequent and life-threatening pathogen in recent years (Pagano et al., 2014; Satlin et al., 2014; Poucha and Satlin, 2017). Previous studies have reported that CRE BSIs account for 4.7% to 23.1% of *Enterobacterales* BSIs in patients with hematologic malignancies in European and American countries (Andria et al., 2015; Satlin et al., 2016). Our data from the hematology department over the past five years shows that the proportion of CRE BSIs was 21.6%, highlighting the need for close attention from hematologists and infection control departments.

In our study, the most common infection sources of CRE BSIs were the respiratory and digestive tracts, which aligns with the typical infection sites in patients with hematologic malignancies (Bodey et al., 1978). Previous researches showed that perianal and multiple infection focuses were the risk factors for BSIs (Giannella et al., 2014). We noticed that perianal infection was an important source of CRE BSIs, and multiple infection focuses were more frequently observed in patients in CRE BSIs compared to CSE BSIs. Thus, we should be vigilant about the possibility of CRE BSIs when patients had these infectious focuses, and CRE screening was necessary for these patients. The infection sources of BSIs are sometimes unclear in patients with hematologic malignancies due to immunodeficiency (Keng and Sekeres, 2013), which would increase the difficulty for hematologists in conducting an appropriate antimicrobial treatment. Our study showed that most CRE BSIs could be traced back to specific infectious sources, which may provide some clues to hematologists.

Previous research has identified several risk factors for CRE infection in patients with hematologic malignancies, including prolonged hospitalization, neutropenia, chemoradiotherapy, allogeneic hematopoietic stem cell transplantation (HSCT), and prior ICU hospitalization (Satlin et al., 2016; Averbuch et al., 2017; Li et al., 2019; Vinker-Shuster et al., 2019; Amanati et al., 2021; Palacios-Baena et al., 2021). Our study found that the majority of CRE BSIs occurred during the neutropenic phase of treatment and that patients with a history of multiple hospitalizations, ICU exposure, and prior CRE infection were at significantly increased risk. Furthermore, CRE BSIs were more frequently observed in patients with uncontrolled diseases compared to those in remission, underscoring the importance of disease status in risk assessment. In terms of laboratory indicators, our data showed that infection-related markers such as CRP, PCT, and IL-6 were elevated in patients with BSIs, but their levels were similar between CRE and CSE BSIs. Therefore, while these markers are sensitive for detecting BSIs, they are not discriminative between CRE and CSE infections.

Monitoring of CRE strain types in hematology ward and WGS of epidemic CRE isolates could provide more in-depth information for hematologist and infection control departments about the CRE BSIs. We noticed that the predominant type of CRE was CRKP in our center, which was consistent with the prevalent CRE strain in Chinese hospitals (Liao et al., 2020). Result of WGS showed that ST11 and *bla*KPC-2 were the major ST type and carbapenemase of CRKP, and almost every strain carried multiple β-lactamase genes and plasmids. Genomic analysis shows that the core-genome alignments of *bla*KPC-2 and *bla*NDM-5 gene in these isolates were quite similar, indicating a high possibility of horizontal transmission within the ward, which required high attention from clinicians. Moreover, it should be emphasized that the mortality rates of patients infected with ST11 strains carrying *bla*KPC-2 were higher than those with other CRE strains. Therefore, identification of the ST and the carbapenemase types carried by CRE strains was of great value in predicting the mode of transmission and prognosis.

Clinically, the broad antibiotic resistance of CRE had posed significant challenges to the antimicrobial treatments. Previous reports had shown patients with CRE BSIs presented high infection-related mortality, especially in patients with hematologic malignancies (Satlin et al., 2016). In our study, infection related septic shock and mortality rate were significantly higher in CRE BSIs compared to CSE BSIs. Currently, polymyxins, ceftazidime/avibactam (CAZ/AVI), tigecycline were recommended for treatment of CRE infections in patients with hematologic malignancies (Doi, 2019; Paul et al., 2022; Zeng et al., 2023), but the efficacy of these drugs in CRE BSIs needs further clarification. Because novel enzyme inhibitor complex antibacterial drugs were not available at our hospital during this research period, the hematologists did not conducted treatment with these agents for CRE BSIs. We found that administration of tigecycline or polymyxins B could reduce mortality, with the combination therapy showing the best therapeutic efficacy. Therefore, combination antimicrobial therapies based on these drugs should be considered for treating CRE BSIs.

It is important to note that the mortality rate of patients with CRE BSIs significantly increases when patients develop septic shock, underscoring the need for appropriate empirical anti-infective therapy to achieve successful outcomes and improve patient survival. However, due to the delay of microbiological culture results in clinical practice, hematologist solely rely on individual experience to choose anti-infective strategies in the past, but sometimes this empirical treatment does not benefit the patient. Taking into account of our research results, empirical antimicrobial strategies should consider patient factors, infection sites, risk factors for CRE BSIs, and the prevalent strains and their corresponding genotypes and enzymotypes in the ward.

Limitations of this study include its single-center design, the lack of availability of novel enzyme inhibitor complex antibacterial drugs during the study period, and the potential timing discrepancy between blood culture specimens and blood samples of inflammatory markers in a small number of patients.

In summary, due to the high mortality rates associated with CRE bloodstream infections (BSIs), hematologists must remain vigilant about this increasingly common infection type. The predominant pathogen in hematology wards is ST11 CRKP carrying *bla*KPC-2. A comprehensive approach that considers the risk factors for CRE BSIs and the microbiological characteristics of the prevalent strains is essential for effective treatment.

## Data Availability

The datasets presented in this study can be found in online repositories. The names of the repository/repositories and accession number(s) can be found below: https://www.ncbi.nlm.nih.gov/genbank/, https://www.ncbi.nlm.nih.gov/bioproject/PRJNA1088820.
